# Efficient removal of 2,4,6-trinitrotoluene (TNT) from industrial/military wastewater using anodic oxidation on boron-doped diamond electrodes

**DOI:** 10.1038/s41598-024-55573-w

**Published:** 2024-02-27

**Authors:** Małgorzata Szopińska, Piotr Prasuła, Piotr Baran, Iwona Kaczmarzyk, Mattia Pierpaoli, Jakub Nawała, Mateusz Szala, Sylwia Fudala-Książek, Agata Kamieńska-Duda, Anna Dettlaff

**Affiliations:** 1 Faculty of Civil and Environmental Engineering, Gdańsk University of Technology, Narutowicza 11/12, 80-233 Gdańsk, Poland; 2 Military Institute of Armament Technology, Wyszyńskiego 7, 05-220 Zielonka, Poland; 3 Faculty of Electronics, Telecommunications and Informatics, Gdańsk University of Technology, Narutowicza 11/12, 80-233 Gdańsk, Poland; 4 Military University of Technology, S. Kaliskiego 2, 00-908 Warsaw, Poland; 5 Faculty of Chemistry, Gdańsk University of Technology, 11/12 Narutowicza Str., 80-233 Gdańsk, Poland

**Keywords:** Environmental chemistry, Environmental impact, Engineering, Materials science

## Abstract

With growing public concern about water quality particular focus should be placed on organic micropollutants, which are harmful to the environment and people. Hence, the objective of this research is to enhance the security and resilience of water resources by developing an efficient system for reclaiming industrial/military wastewater and protecting recipients from the toxic and cancerogenic explosive compound—2,4,6-trinitrotoluene (TNT), which has been widely distributed in the environment. This research used an anodic oxidation (AO) process on a boron-doped diamond (BDD) electrode for the TNT removal from artificial and real-life matrices: marine water and treated wastewater. During experiments, TNT concentrations were significantly decreased, reaching the anodic degradation efficiency of above 92% within two hours and > 99.9% after six hours of environmental sample treatment. The presented results show the great potential of AO performed on BDD anodes for full-scale application in the industry and military sectors for TNT removal.

## Introduction

Commencing in the nineteenth century, chiefly driven by armed conflicts like the First and Second World Wars, there was a substantial increase in the mass production of nitro-organic explosive compounds^1^. Among them, 2,4,6-trinitrotoluene (TNT) was the most widely used due to its cost-effective production and low sensitivity to shock and friction, which reduces the possibility of an unexpected detonation^2,3^. However, the widespread use of TNT increases the risks of environment contamination. TNT is categorised as a class C human carcinogen^4^, and the United States Environmental Protection Agency (US EPA) has classified TNT as a contaminant of emerging concern (CEC), which has resulted in 2,4,6-trinitrotoluene being routinely monitored, and being found at low levels in many water sources. Even at these low levels it may cause may cause ecological or human health impacts^5^. Additionally, there is evidence showing that explosive materials and their by-products are being accumulated in the marine food chain, which in turn affects human food chains^6,7^.

2,4,6-trinitrotoluene is still being produced and used for military and commercial applications. In the course of manufacturing and processing, the generation of wastewater occurs, wherein the concentration of TNT may reach up to 160 mg L^−1^^8–10^. Moreover, a single 2,4,6-trinitrotoluene ammunition factory can produce 1,900,000 dm^3^ of explosive-contaminated effluent per day^3^. Historically, “pinkwater” (or “redwater”), the effluent containing mainly TNT, was often released directly into lagoons^10^. Presently, numerous countries are tightening regulations concerning the quality of wastewater released into the environment. Commonly used wastewater treatment methods, such as biological and physicochemical approaches, have various drawbacks. Firstly, there is a limitation in the microbiological applicability, especially in the presence of other xenobiotics, which may result in inadequate treatment. Secondly, the physical processes such as filtration, reverse osmosis, and adsorption separate contaminants rather than degrade them, thereby generating another waste contaminated with explosive compounds (at a higher concentration) that still requires treatment^8,9,11^.

Despite the hazards associated with the presence of TNT in the environment, in the newest Directive (EU) 2020/2184 on the quality of water intended for human consumption^12^, there is no information regarding TNT acceptable levels. Similarly, established “Watch List” regulations (Watch List no. 3^13^, Directive 2013/39/EU^14^) requiring countries to monitor selected contaminants in water do not include explosive materials. So far, only the US EPA has assessed the risks associated with the presence of TNT in drinking water. Suggesting a limit of 0.1 mg L^−1^, for which, an estimated lifetime cancer risk is 1 in 10,000^15^. Nevertheless, we believe efforts should focus on sustaining TNT concentrations below the 2 μg L^−1^ recognized as a safe dose^15^. Thus, the authors propose this level as a recommended drinking water limit for EU countries. To achieve this concentration level, a cost effective and scalable wastewater treatment method is required.

Anodic oxidation (AO) is a method that exhibits potential in effectively eliminating persistent organic compounds. This technique encompasses the indirect oxidation of organic pollutants through oxidizing agents and/or direct oxidation at the anode surface^16,17^. Among the anodic materials used in AO processes, boron-doped diamond (BDD) is distinguished its excellent resistance to fouling, and ability to withstand extreme conditions (high potentials, high temperature/pressure, corrosive environments)^18–20^. Furthermore, using BDD in AO enables to direct production of a strong oxidising agent, the hydroxyl radical (·OH), via reaction (1)^21^. Compared to other anode materials, BDD exhibits a higher kinetic overpotential of O_2_ generation, which reduces the competition from oxygen evolution reaction, and increases the efficiency of ·OH production. The hydroxyl radical is known for its ability to mineralize a wide variety of organic species, including persistent organic pollutants^21–23^. Furthermore, BDD is weakly interacting with generated ·OH radicals so that most of the generated oxidant can react with the pollutants available in the solution instead of being chemisorbed at the electrode^21^. In addition to the hydroxyl radical, other oxidising compounds can also be formed according to the Eqs. (1–6), which may improve the oxidation process^24,25^.

1 $$\cdot OH \to {O}^{\cdot }+ {H}^{+} +{e}^{-}$$

2 $$2\cdot OH \to {{H}_{2}O}_{2}$$

3 $${{H}_{2}O}_{2} \to {O}_{2}+ {2H}^{+} +{2e}^{-}$$

4 $${2O}^{\cdot } \to {O}_{2}$$

5 $${O}_{2} +{O}^{\cdot }\to {O}_{3}$$

6 $$\cdot OH+ {{H}_{2}O}_{2}\to {HO}_{2}^{\cdot }+{H}_{2}O$$

In this work, we present for the first time the effective anodic oxidation of TNT using BDD electrodes, together with an analysis of the by-products of this process. The research involved testing the efficiency of TNT removal depending on solution matrix. Studies were carried out in both laboratory-grade purity matrices and environmental samples, which were selected to investigate removal efficiency in matrices where higher concentrations of TNT can be expected: seawater and samples from wastewater treatment plants. The TNT removal process was controlled by applying two techniques in parallel: (i) liquid chromatography with a photodiode array detector and (ii) electrochemical sensing. An analysis of the by-products generated during the process was also carried out using gas chromatography-tandem mass spectrometry. An analysis of energy consumption was also conducted, which could support the possibility of introducing this method into industry. The presented work is a first step towards the application of the AO method on BDD in explosives removal from aquatic environments.

## Results and discussion

### Anodic oxidation of TNT-spiked samples

**Figure 1:**
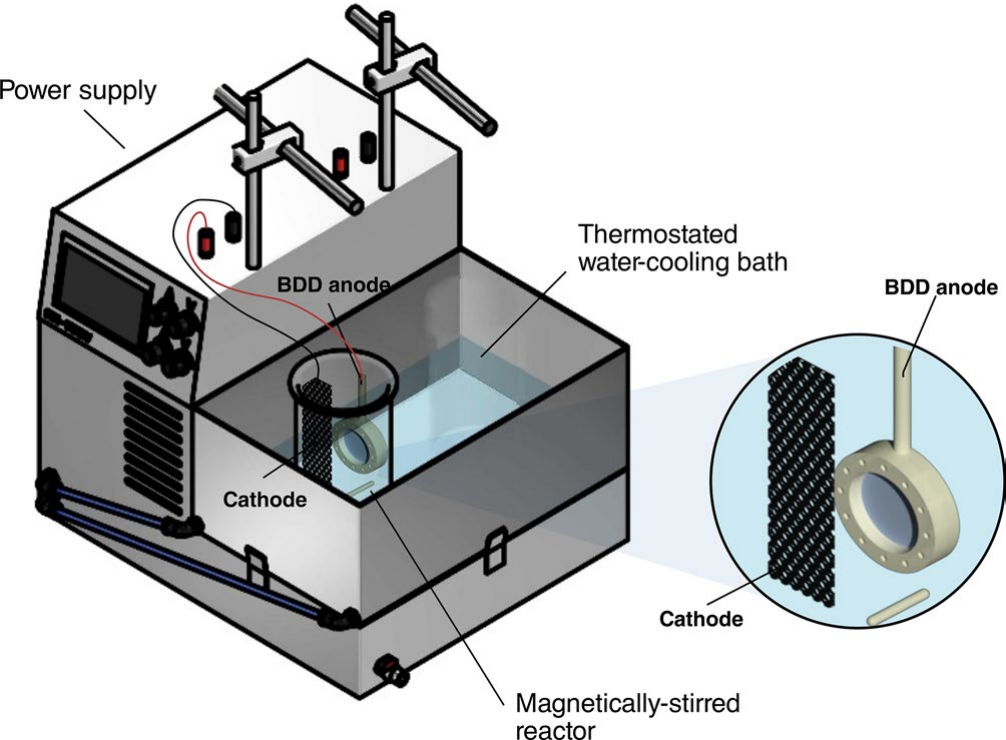
Advanced oxidation setup (batch reactor) scheme using BDD anode and stainless-steel cathode in the undivided electrolytic cell.

The AO experiments were performed using a BDD electrode as an anode and stainless-steel mesh as a cathode. The electrodes were placed in a 500-mL undivided electrolytic cell, which was put in a specially designed experimental set-up consisting of a cooling bath and magnetic stirrer (Fig. 1). The anode geometric surface area was 10.5 cm^2^. The distance between them was kept constant at about 2.5 cm. All AO tests were performed under galvanostatic conditions, with a current density of 50 mA cm^−2^, which was adjusted to the treated wastewater sample conductivity, which was the lowest among studied matrices.

The ability to remove aromatic compounds was first tested in an uncomplicated, laboratory-grade purity matrix of 0.1 M phosphate buffer solution (**0.1M_PBS)**. Next, TNT degradation was investigated in real-life samples—treated wastewater (**TWW**) and Baltic Sea marine water (**MW**) (full characterisation of TWW and MW samples can be found in SI 2). Considering the elevated concentration of chlorides in the real-life samples, the decision was made to broaden the scope of the research to examine the impact of chlorides on the effectiveness of the treatment process. For this purpose, NaCl was dissolved in the PBS solution to obtain a solution with a concentration of 100 mg Cl^−^ L^−1^ (**0.1M_PBS_100mgCl**) and 200 mg Cl^−^ L^−1^ (**0.1M_PBS_200mgCl)**. Each AO process was carried out for 8 h at a temperature of 20 ± 3 °C, and the solutions were spiked with TNT stock solution resulting in the same initial concentration of 50 mg L^−1^ (50 ppm).

**Figure 2:**
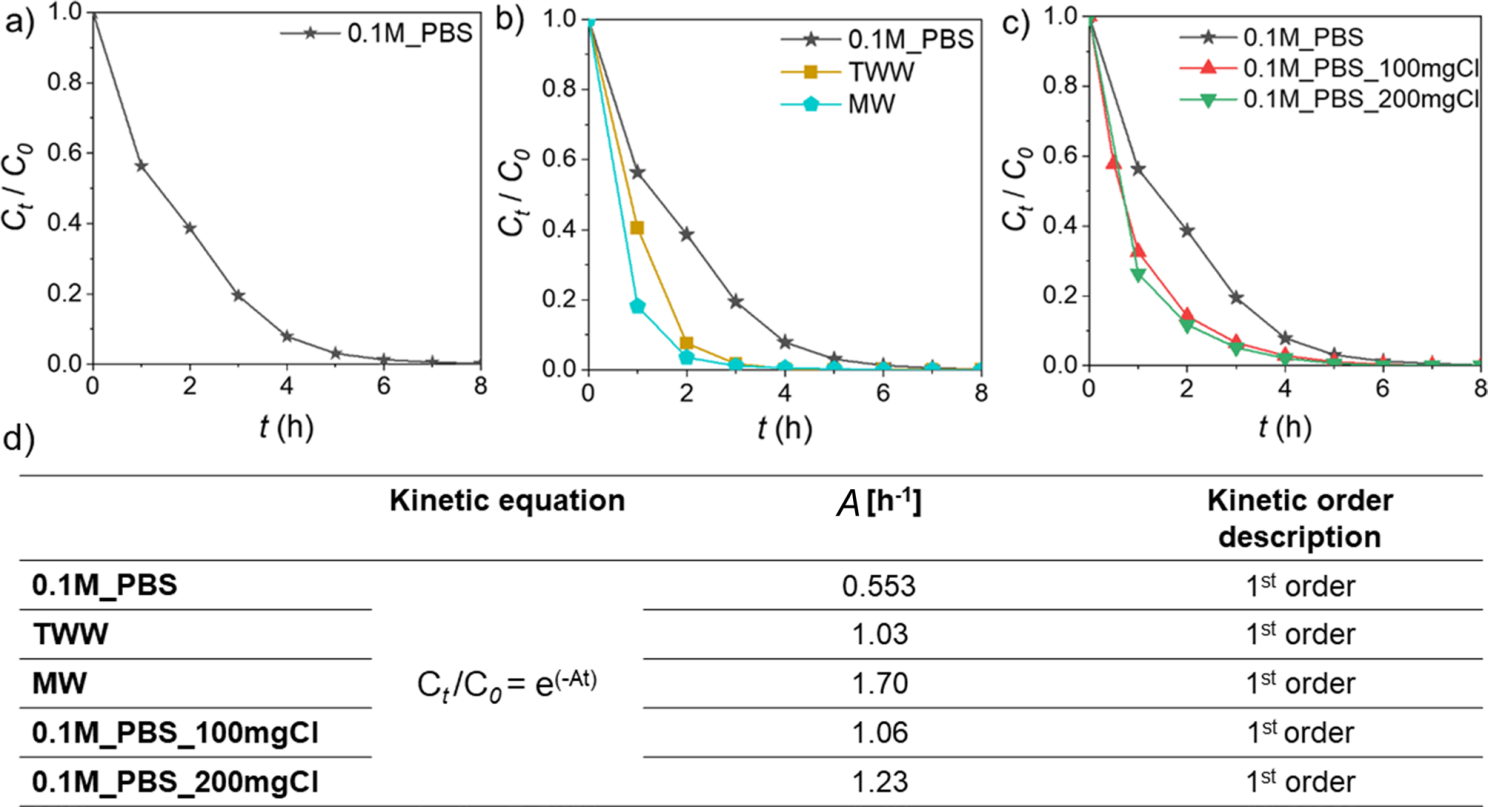
Trend with a time of oxidising TNT, normalised concerning the initial concentration (*C*_*0*_ = 50 mg L^−1^) based on HPLC–PDA results in (**a**) 0.1 M PBS, (**b**) saline artificial samples: 0.1M_PBS_100mgCl and 0.1M_PBS_200mgCl, (**c**) environmental samples: TWW and MW (0.1M_PBS sample is left as reference), (**d**) kinetic order description and kinetic equation of TNT removal rate (*A*—reaction rate constant [h^−1^], *t*—time [h]).

Anodic oxidation of TNT-polluted samples was first conducted in the PBS solution. Phosphate buffer of pH 7.4 was prepared to mimic the conditions of wastewater treatment plant effluents^26^. Chloride-free electrolytes as phosphate buffer support electrogeneration of reactive oxygen species including ·OH, H_2_O_2_, O_3_, or O^2−^^27^, and do not have a supportive effect on the anodic oxidation, as it would be expected in other electrolytes containing chloride or sulphate ions^28^. The efficiency of TNT removal by AO is presented as the concentration (determined by the HPLC–PDA technique) as a function of time (Fig. 2a). As can be seen, with increasing time the concentration successively decreases reaching the value of normalised concentration *C*_*t*_/*C*_*0*_: 0.56, 0.08, and 0.006 after 1 h, 4 h, and 6 h, respectively. Furthermore, after 8 h of AO, 99.8% of initial TNT was degraded reaching the value of 93 µg L^−1^.

As TNT degradation products cannot be identified using the HPLC–PDA method, select samples have been analysed using a GC–MS/MS technique. The gas chromatography results confirmed a significant decrease in the concentration of TNT in the spiked samples (see Table 1). After 8 h treatment, a concentration of 86 µg L^−1^ was achieved, which agrees with previous HPLC–PDA results. The concentration is anticipated to continue decreasing; however, further investigation is needed to access the total TNT mineralisation. This significant reduction in the concentration of TNT indicates a high efficiency of removing this compound by anodic oxidation on the BDD electrode.

**Table 1:** TNT and its degradation products, identified in 0.1 M PBS using GC–MS/MS in selected reaction monitoring mode.

Matrix	0.1M_PBS			
Treatment time	0 h	4 h	8 h	Blank
TNT / mg L^−1^	49.6	4.04	0.086	nd
TNB / mg L^−1^	nd	0.021	nd	nd
1,3-DNB / mg L^−1^	nd	0.016	0.016	nd
4-NT / mg L^−1^	nd	0.011	0.011	nd
4-A-2,6-DNT / mg L^−1^	nd	0.149	0.065	nd
2,6-DNT / mg L^−1^	nd	0.044	0.033	nd
2-A-4,6-DNT / mg L^−1^	nd	0.055	0.181	nd
2,4-DNT / mg L^−1^	nd	0.034	0.024	nd

*nd* not detected.

The limit of detection and quantification for GC–MS/MS in the selected reaction monitoring mode used for the analysis of TNT and its degradation products are shown in Tables S5, S6, SI 3.

**Figure 3:**
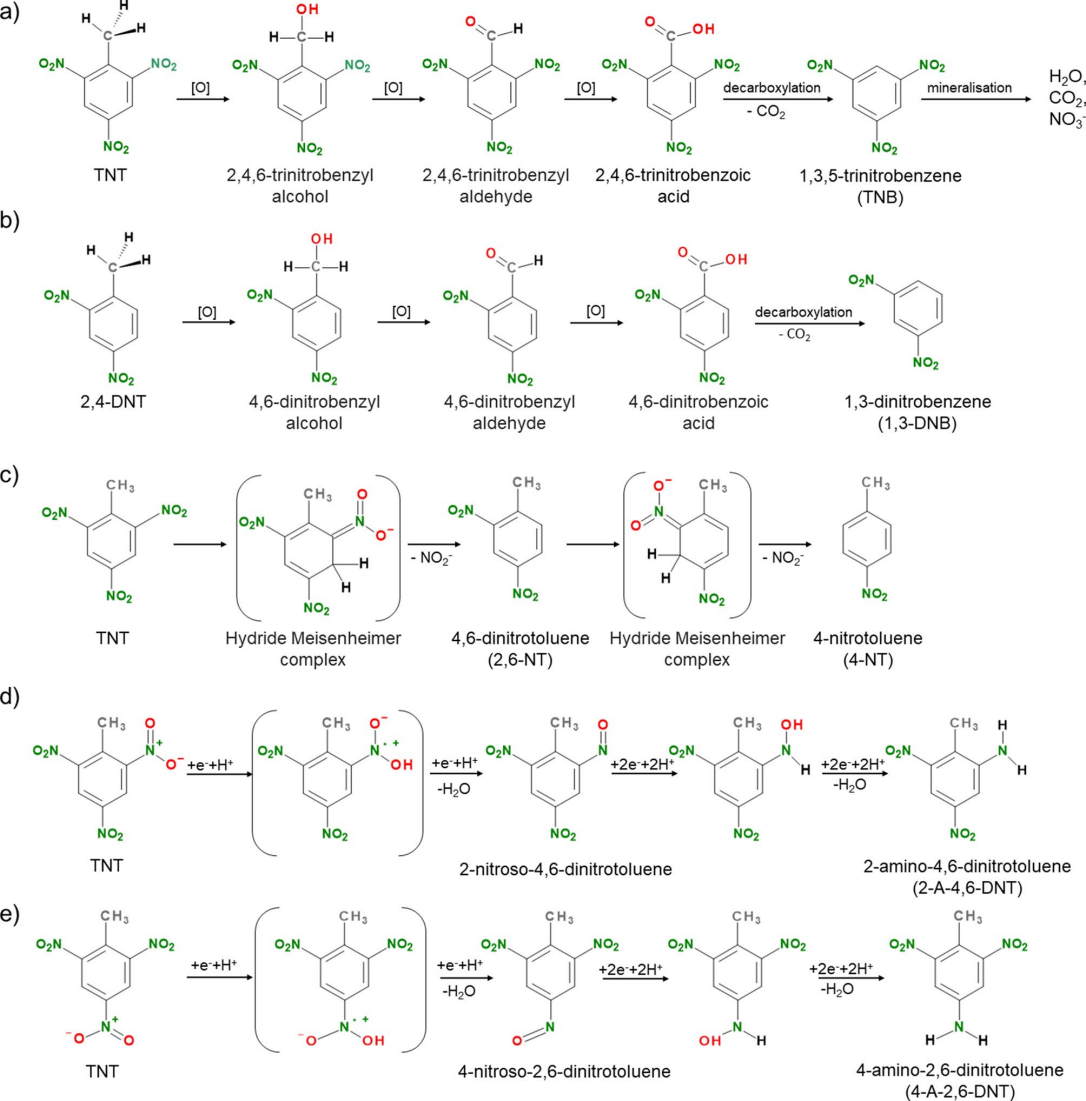
Utilizing identified intermediates, proposed pathways for (**a**) electrooxidative decomposition of TNT, and (**b**) 2,4-DNT; (**c**) 2,4-DNT and 4-NT formation through TNT-Meisenheimer complex formation; (**d**) creation of (**d**) 2-amino-4,6-dinitrotoluene, (**e**) 4-amino-2,6-dinitrotoluene via electroreduction.

The chromatography results indicated the formation of TNT oxidation intermediates such as 1,3,5-trinitrobenzene (TNB) and 1,3-dinitrobenzene (1,3-DNB). The appearance of the 21 µg L^-1^ TNB in the 0.1M_PBS sample after 4 h of the AO process and its absence after 8 h agrees with the mechanism of TNT decomposition shown in Fig. 3a. The proposed oxidation process is a chain of H-abstraction reactions. First, a methyl group is oxidised to an aldehyde moiety –CHO, which in turn is transformed into a carboxyl –COOH group. Next is the decarboxylation process that leads to the TNB compound. Then, with time, the full demineralization of the TNB to water, carbon dioxide and nitrate ions could be is expected^29,30^. With the increasing duration of the AO process, there is a noticeable decrease in the pH of the reaction mixture from pH 7.77 to 7.06 and from 7.4 to 7.18 (Table S4, SI 3) in the samples with low Cl^-^ influence: 0.1 M PBS and TWW, respectively. The decrease in pH could be explained by the increasing concentration of nitrate ions in the presence of week-base cations and/or short-chain carboxylic acids evolution during TNT degradation^31^.

1,3-DNB is in turn formed from the oxidation of 2,4-dinitrotoluene (2,4-DNT) (Fig. 3b). The presence of dinitrotoluenes (2,4-DNT and 2,6-dinitrotoluene—2,6-DNT) and 4-nitrotoluene (4-NT) may be explained by the formation of hydride Meisenheimer complexes^32–34^ (Fig. 3c). The electronegative nitro groups extract the π electrons from the aromatic ring of TNT, thereby rendering the aromatic nucleus electrophilic and enabling nucleophilic attack. In this way, it is possible to form a negatively charged nonaromatic complex, which can be rearomatized after -NO_2_ release forming dinitrotoluenes^34^.

Apart from the electrooxidation products, Table 1 also displays products obtained through the TNT reduction process. The presence of 4-amino-2,6-dinitrotoluene (4-A-2,6-DNT) and 2-amino-4,6-dinitrotoluene (2-A-4,6-DNT) can be explained by the cathodic reaction taking place at the stainless-steel counter electrode (Fig. 3d,e). It should be noted that although only the diamond anode was polarised, current must also be passed through the cathode to complete the cell. While 4-A-2,6-DNT and 2-A-4,6-DNT exhibit lower toxicity compared to TNT^35^, their presence is still linked to health risks. However, it should be emphasized, that identified TNT degradation by-product concentrations are almost 100 times lower (< 0.2 mg L^−1^) than the initial concentration of TNT (50 mg L^−1^). To avoid the formation of electroreduction products, (1) the anode and cathode could be separated by a membrane or (2) different cathode material can be evaluated, and (3) different advanced oxidation processes can be merged.

To explore the impact of chloride anions, present in environmental samples on the anodic oxidation process, the analyses were also performed in chloride-spiked PBS (100 and 200 mg Cl^−^ L^−1^). The results of the HPLC–PDA investigation of the saline artificial samples are presented in Fig. 2b. During the first hour of treatment *C*_*t*=*1*_/*C*_*0*_ decreases to 0.33 and 0.26 for 0.1M_PBS_100mgCl and 0.1M_PBS_200mgCl samples, respectively. The rate of explosive material decomposition in 0.1M_PBS_200mgCl solution is more than double compared to PBS-only sample. After 3 h of treatment, *C*_*t*=*3h*_/*C*_*0*_ was recorded below 0.1 for both salty solutions (*C*_*t*=*3h*_/*C*_*0*_ = 0.066 for 0.1M_PBS_100mgCl, and *C*_*t*=*3h*_/*C*_*0*_ = 0.052 for 0.1M_PBS_200mgCl), whereas in pristine 0.1M_PBS the *C*_*t*_/*C*_*0*_ value reached almost 0.2 (*C*_*t*=*3h*_/*C*_*0*_ = 0.195). Moreover, after 7 h of AO, the concentration of TNT recorded for 0.1M_PBS_200mgCl was below the limit of detection (LOD) value of the HPLC–PDA apparatus (LOD = 2.9 µg L^−1^).

The results show that a saline environment enhances the rate of nitroaromatic TNT degradation over time. A similar effect was also observed by Kuo et al.^36^. The effect can be explained by the presence of active chlorine species originating from the chloride ions oxidation to active chlorine species like dissolved (Cl_2,aq_), hypochlorous acid (HClO) and hypochlorite ion (ClO^−^) via reactions (Eqs. 7–9)^23^. The acceleration of the degradation of aromatic compound pollution may be attributed to the presence of active chlorine species.

7 $$2{Cl}^{-}\to {Cl}_{2,aq}+2{e}^{-}$$

8 $${Cl}_{2,aq}+{H}_{2}O\to HClO+{H}^{+}+{Cl}^{-}$$

9 $$HClO\underset{ }{\leftrightarrow }{ClO}^{-}+ {H}^{+}$$

The electrooxidation process was then carried out in a complex, real-life environmental matrices. For this purpose, the measurements were made in samples collected from the Baltic Sea (MW) and a wastewater treatment plant (TWW). The results of the HPLC–PDA investigation of TWW and MW are presented in Fig. 2c. Normalized concentration *C*_*t*_/*C*_*0*_ in TWW matrix was 0.4, 0.004 after 1 h and 4 h of treatment, respectively; and in Baltic Sea marine water matrix: 0.18, 0.006 after 1 h, and 4 h, respectively. After 6 h of AO, for both environmental solutions the value of *C*_*t*=*6h*_/*C*_*0*_ was below LOD of HPLC–PDA which, when converted, gives > 99.9% removal of TNT. Compared to the 0.1M_PBS sample, environmental matrices have a positive effect on the nitroaromatic compounds' degradation rate. After 1 h of anodic oxidation, 42%, 61%, and 80% of the initial concentration of TNT were reduced for 0.1M_PBS, TWW and MW matrices, respectively. After only two hours of treatment, TNT removal efficiencies were 92% for TWW and 96% for MW, while electro-assisted advanced oxidation processes *e.g.*, combined electrolytic and H_2_O_2_ system led to only 70% removal of TNT in the same timeframe^37^.

In all studied matrixes TNT removal represents 1st kinetic order (Fig. 2d), where the highest reaction rate constant, *A*, is noticed in the marine water matrix (*A* = 1.23 h^−1^) and under these conditions we are observing the fastest initial disappearance of the analyte. Hence, reaction rates analysis also confirms that saline environment enhances the TNT AO removal.

It is worth noting, that well-conductive matrices, with conductivity above 10 mS cm^−1^, showed reduced energy consumption in comparison to the less-conductive solution of treated wastewater (conductivity < 2 mS cm^−1^) (for details please see Table S4, SI 3). During 8 h of treatment using anodic oxidation on BDD energy consumption needed for the AO process varied between 68.0 and 84.8 kWh m^-3^ for most tested samples (only the TWW environment generates consumption of 281.6 kWh m^−3^). The energy consumption associated with the electrochemical oxidation of organic micropollutants is contingent upon various factors such as the type of anode, current density, reaction time, and the structure of the micropollutant. This complexity makes it challenging to directly compare the resulting energy consumption data with existing literature. However, referring to our previous work^38^, where BDD anode was also applied for the removal of forever chemicals (polyfluorinated alkyl compounds) from landfill leachates, energy consumption varied between 88 to 114 kW hm^−3^ after 8 h of AO (*j* = 75 mA cm^−2^). In comparison to our other study^39^, the energy consumption at the specific current 50 mA cm^−2^ after 8 h of treatment was proportional to present results regarding the saline environment. This confirms that electrochemical oxidation is the preferable technique for more complex matrices with higher conductivity.

The presented results show potential for the application of AO in the “pink water” treatment. It should be emphasized that the presented method can be operated without introducing additional species such as iron catalyzers or hydrogen peroxide/oxide streams often used in other purification methods (see Table 2). The technique is robust, easy to operate and flexible through electrode material modification, and current adjustment in case of fluctuating load of contaminants in the wastewater streams.

**Table 2:** Comparison of TNT removal methods in aqueous matrices.

Method	Conditions	c_TNT_/mg L^-1^	Matrix	Sample volume/mL	Control technique	Time/h	TNT removal/%	Analysis of by-products	Ref.
Electrolysis and H_2_O_2_	Voltage = 10 V, H_2_O_2_ = 300 mg L^−1^, Fe^2+^ = 2.52 mmol L^−1^	149 – 202	Wastewater ammunition destroyed scrap	700	UV–vis spectrophotometer	2	~ 70	no	^37^
Ultrasonic irradiation combined with UV/TiO_2_	Ultrasonic power = 110 W cm^−2^, T = 288 K, UV intensity = 96 W, TiO_2_ = 3000 mg L^−1^, O_2_ = 300 mL min^−1^	350 (DNT and TNT)	Industrial wastewater	700	TOC analyser equipped with UV reactor and NDIR detector	8	~ 99	yes	^40^
Adsorption on Fe/SiO_2_ nanocomposite	Room temperature	50	DI water	100	UV–vis spectrophotometer	nd	24.8	yes	^41^
Combined zero-valent iron and Fenton processes	0.8 g of wool metallic iron, pH = 3.0, Fe^2+^ = 100 mg L^−1^, H_2_O_2_ = 500 mg L^−1^	156	Industrial wastewater	250	HPLC–PDA	0.5	100	no	^42^
Electro-Fenton treatment	*j* = 55 mA cm^−2^, 0.2 mM Fe^2+^, pH = 3.0	45	NaClO_4_ + HClO_4_	250	HPLC–ESI–MS/MS	0.33	99	yes	^31^
Photocatalytic with TiO_2_ –borosilicate glass	UV intensity = 125 W	2	Diluted industrial wastewater	400	UV–vis spectrophotometer	2	80	no	^43^
Advanced oxidation	*j* = 50 mA cm^−2^	50	Marine water, wastewater sample	500	HPLC–PDA	2	> 92	yes	This work
						6	> 99.9		

Nevertheless, in line to the circular economy, a holistic approach for efficient wastewater management is needed. The presented technology has a great potential for modular application as well for integrated treatment (together with other treatment processes). Moreover, in the full-scale application it may be used for any type of wastewater stream treatment (inc. main wastewater stream, any by-pass stream or concentrate obtained after reverse osmosis or nano-/ultra-filtration). The application as a fit-of-purpose manner is coherent with EU Green Deal ambitions^44^, (e.g., Towards Zero Pollution, Water-Smart Society) and UN Sustainable Development concepts^45^. Such an approach will help to close the water cycles at industrial production sites. This in fact would decouple industrial growth from resource consumption. Moreover, all the circular economy policy would significantly reduce the input of chemical compounds into the environment contributing to an improved water supply for human beings.

### Electrochemical detection of TNT and its by-products before and after AO

TNT removal control was also carried out by electrochemical sensing. While most analyses of effluent compounds in the aqueous environment are performed using chromatographic methods, electrochemical sensors provide a low-cost substitute, as well as enabling on-line control of the process. For this purpose, samples before and after electro-oxidation were tested using electrodes with a high *sp*^*2*^ carbon content, the presence of which increases the sensitivity of the sensor (TNT easily adsorbs onto π-donor graphene surfaces, unlike BDD)^46,47^. Ability of 2,4,6-trinitrotoluene detection on boron-doped diamond/few-layer graphene nanowall electrodes has previously been published in our previous work^46^. Limit of detection for TNT is 73 ppb, whereas the sensitivity is 36.25 µA cm^−2^ ppm^−1^.

**Figure 4:**
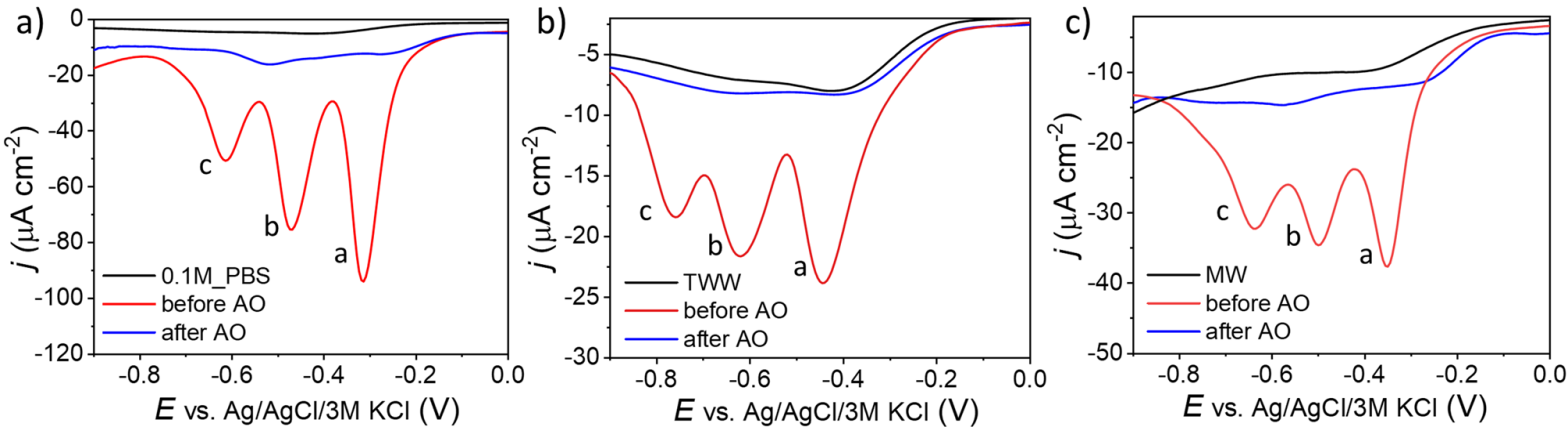
Differential pulse voltammetry made before and after electrooxidation process recorded on BDGNW electrode immersed in (**a**) 0.1 M PBS, (**b**) marine water, (**c**) treated wastewater.

Electrochemical detection was performed by the differential pulse technique (Fig. 4). The measurements were first provided in blank solutions (0.1M_PBS, MW, TWW), and then the sample with spiked 50 mg L^−1^ TNT were tested. The last step was an electrochemical investigation of the solutions after AO.

It was found that TNT is presented in all the samples before the AO process provide three well-separated peaks observed at negative potentials: − 0.31 V (peak “a”), − 0.47 V (peak “b”) and − 0.61 V (peak “c”) in 0.1M_PBS, − 0.35, − 0.50 and − 0.63 V in marine water, and − 0.44, − 0.62 and − 0.76 V in treated wastewater *vs.* Ag/AgCl/3 M KCl. The reduction signals are observed in the potential range typical for the reduction of nitro groups^46,48,49^. The reduction of TNT is a multistep process as the molecule contains three electroactive nitro (–NO_2_) moieties (oxygen and nitrogen atoms building –NO_2_ group are highly electronegative, which causes strong polarization of –N–O bond, making nitro moiety easily reducible^1^). Hence, each of the peaks corresponds visible in Fig. 4 corresponds to the individual reduction of –NO_2_ group to amine (–NH_2_) moiety^46^. The peaks in environmental samples are shifted towards more negative potentials, which may be caused by their lower pH value compared to PBS (the reduction process heavily depends on pH^46^). Then the samples after electrooxidation were tested. It should be noted that after AO there is no evidence of the explosive materials in all the investigated samples (the values are below LOD).

## Conclusions

The efficacy of anodic oxidation in breaking down TNT has been demonstrated at the laboratory scale in aquatic environments employing BDD electrodes. Our results show that the AO process using diamond electrodes is very effective towards TNT degradation (> 92% removal within 2 h and above 99.9% within 6 h in environmental matrices). In each tested matrix, the anodic oxidation caused a significant decrease of explosive material reaching concentration below 0.1 mg L^−1^.

The research presented here is a first step towards the application of the AO method using BDD to remove explosives from aquatic environments. Our results show that electrochemical oxidation is the preferable technique for more complex matrices with higher conductivity. This technique creates a route for water reuse (water circular economy approach) in the industrial and military sectors. Employing AO process for wastewater treatment proves particularly advantageous for factories engaged in explosives production, mitigating the significant environmental pollution caused by these compounds. Furthermore, suitable, and sustainable technology for the backwash salt water (potentially generated during the chemical weapon removal in the Baltic Sea shore), is suggested to purify saline water contaminated with explosive compounds.

The anodic oxidation process on the BDD anode is promising for direct treatment of wastewater from the TNT. Given high removal efficiency AO process on BDD shows great potential for full-scale application, hence process optimisation, scalability, operation, and maintenance cost need to be studied further for its actual field application.

## Methods

### Fabrication and characterisation of BDD electrodes

Following the previously reported synthesis procedure^50^, all the BDD electrodes were fabricated by microwave plasma-assisted chemical vapour deposition system (SEKI Technotron AX5400S, Japan) on an Nb substrate. Before deposition, the substrates were subjected to surface roughening by sandblasting using a compressed air sandblasting gun and corundum sand, cleaned in analytical-grade acetone and isopropanol in an ultrasonic bath, and seeded in a water-based nanodiamond slurry. The growth conditions were: substrate temperature of 700 °C, microwave power of 1300 W, and process duration of 12 h. The boron doping level expressed as the [B]/[C] ratio in the gas phase, was 2000 ppm. No post-treatment cleaning procedure was performed on the electrodes. Electrodes preparation and characterisation (scanning electron microscopy, Raman spectroscopy, electrochemical behaviour) details are presented in SI 1.

### Chemicals and solutions

TNT was obtained by the chemical plant “NITRO-CHEM S.A.” and purified for testing purposes by the Military Institute of Armament Technology. The purification process consisted of 3 recrystallizations from acetone and drying until a constant weight was measured. The selected properties of the TNT used in the tests are presented in Table S2, SI 2. A stock solution of TNT, in the concentration of 25 g L^−1^, was prepared using 625 mg of TNT dissolved in a 25-mL volumetric flask using acetonitrile (gradient grade for HPLC, purchased from ChemSolve). The remaining chemicals are described in SI 2.

### AO effectiveness control

Chemical analysis, including measurements of pH, conductivity, oxidation reduction potential (ORP), voltage and specific electrical charge, was performed during the electrooxidation process to monitor the anodic oxidation (Table S4, SI 3). For this purpose, 2 mL of solution was sampled every hour (except experiments with real matrix, then 4 mL was sampled). pH and ORP were measured *in-situ*. The energy consumption (ECon) was calculated using Eq. (10)^51^ and expressed in kWh m^−3^.

10 $$ECon=Q\cdot U= \frac{j \cdot A\cdot t }{V}\cdot U,$$

where *Q* corresponds to specific electrical charge [kAh m^−3^], *U* reflects the average cell voltage [V], *j* applied current density [A cm^−2^], *A* is geometric electrode area [cm^2^], *t* refers to the electrolysis time, and *V* is the volume of the electrolyte [m^3^].

High-performance liquid chromatography with a photodiode array detector, HPLC–PDA, (Prominence-i LC-2030 3D plus, Shimadzu, Japan) and electrochemical sensing were used to analyse the effectiveness of TNT removal during the AO process. HPLC–PDA details are shown in SI 3. Electrochemical sensing (ES) was conducted using *sp*^*2*^-rich boron-doped diamond/few-layer graphene nanowall electrodes (BDGNW). BDGNW electrodes were deposited following the previously reported synthesis^46^. Fabrication and characterization of BDGNW are shown in SI 4. ES was performed using differential pulse voltammetry (DPV). The DPV parameters (*p*_*H*_ = 25 mV, *p*_*W*_ = 200 ms, *s*_*t*_ = 500 ms, and a scan rate of 10 mV s^−1^) were based on our previous work^46^.

Gas chromatography equipped with a tandem mass spectrometer (GC–MS/MS) was utilized to detect by-product compounds formed during electro-oxidation. Details can be found in SI 3.

## Supplementary Information

Supplementary Information.

## Data Availability

Correspondence and requests for materials should be addressed to A.D.
