# Author Correction: Efficient removal of 2,4,6-trinitrotoluene (TNT) from industrial/military wastewater using anodic oxidation on boron-doped diamond electrodes

**DOI:** 10.1038/s41598-024-67935-5

**Published:** 2024-07-30

**Authors:** Małgorzata Szopińska, Piotr Prasuła, Piotr Baran, Iwona Kaczmarzyk, Mattia Pierpaoli, Jakub Nawała, Mateusz Szala, Sylwia Fudala-Książek, Agata Kamieńska-Duda, Anna Dettlaff

**Affiliations:** 1 Faculty of Civil and Environmental Engineering, Gdańsk University of Technology, Narutowicza 11/12, 80-233 Gdańsk, Poland; 2 Military Institute of Armament Technology, Wyszyńskiego 7, 05-220 Zielonka, Poland; 3 Faculty of Electronics, Telecommunications and Informatics, Gdańsk University of Technology, Narutowicza 11/12, 80-233 Gdańsk, Poland; 4 Military University of Technology, S. Kaliskiego 2, 00-908 Warsaw, Poland; 5 Faculty of Chemistry, Gdańsk University of Technology, 11/12 Narutowicza Str., 80-233 Gdańsk, Poland

Correction to: *Scientific Reports* 10.1038/s41598-024-55573-w, published online 27 February 2024

The original version of this Article contained an error in the spelling of the author Sylwia Fudala-Książek which was incorrectly given as Sylwia Fudała-Książek.

The original Article has been corrected.

