# Supplementary material for: Efficient removal of 2,4,6-trinitrotoluene (TNT) from industrial/military wastewater using anodic oxidation on boron-doped diamond electrodes
Source: Sci Rep. 2024 Feb 27;14:4802. doi: 10.1038/s41598-024-55573-w (PMC12441148; doi:10.1038/s41598-024-55573-w)
Supplement: Supplementary file 1 — Supplementary Information. [file 41598_2024_55573_MOESM1_ESM.pdf]

Supplemental Information

Efficient Removal of 2,4,6-Trinitrotoluene (TNT) from Industrial/Military Wastewater Using Anodic Oxidation on Boron-Doped Diamond Electrodes

Małgorzata Szopińska[a], Piotr Prasuła[b], Piotr Baran [b], Iwona Kaczmarzyk[c], Mattia Pierpaoli[c], Jakub Nawała[d], Mateusz Szala[d], Sylwia Fudała-Książek[a], Agata Kamieńska-Duda[b], Anna Dettlaff[e,c]*

[a]*Gdańsk University of Technology, Faculty of Civil and Environmental Engineering, Narutowicza 11/12, 80-233 Gdańsk, Poland*

[b]*Military Institute of Armament Technology, Wyszyńskiego 7, 05-220 Zielonka, Poland*

[c]*Gdańsk University of Technology, Faculty of Electronics, Telecommunications and Informatics, Narutowicza 11/12, 80-233 Gdańsk, Poland*

[d]*Military University of Technology, S. Kaliskiego 2, 00-908 Warsaw, Poland*

[e]*Gdańsk University of Technology, Faculty of Chemistry, 11/12 Narutowicza Str., 80-233, Gdańsk, Poland*

[*]*Corresponding Author e-mail address: anna.dettlaff@pg.edu.pl*

Table of Contents

SI 1: Fabrication and characterisation of BDD electrodes

SI 2: Chemicals and solutions

SI 3: Anodic oxidation process control

SI 4: Fabrication and characterization of BDGNW electrodes

References

SI 1: Fabrication and characterisation of BDD electrodes

Characterisation techniques

SEM: Scanning electron microscopy (SEM) images were acquired by a Phenom XL microscope, using a 10-kV beam accelerating voltage, working in high vacuum mode, equipped with a secondary electron detector (SED).

Raman: Raman spectra were acquired using LabRam ARAMIS (Horiba Scientific) using a 532 nm laser. A CCD detector cooled to -20°C using thermoelectric cooling was employed, with an integration time of 5 seconds (20 averages). The diffraction grating utilized had a density of 300 lines per millimetre.

Electrochemical measurements: To explore the electrochemical properties of the BDD electrodes, cyclic voltammetry (CV) was performed using a potentiostat-galvanostatic (VMP-300, BioLogic, France) equipped with the EC-Lab software. The measurements were conducted in a three-electrode cell setup. For the counter electrode, a platinum wire was utilized, while a reference electrode of Ag/AgCl/3M KCl was employed. Cyclic voltammetry was first performed in phosphate buffer solution (PBS), and secondly in 1 mM $[Fe(CN)_6]^{3-/4-}$ in 1 M KCl solution. Before the electrochemical measurements, the solutions underwent deoxygenation with Ar gas assuming 1 min for each 1 mL of solution.

BDD electrodes characterisation: The surface analysis made by SEM for polycrystalline BDD layers deposited on Nb showed well-faceted crystallites (Fig. S1a, SI 1). Niobium substrate is uniformly covered by BDD coating. The size of the grains is in the range of 0.5 – 3 µm, which classifies the boron-doped diamond as a microcrystalline material. Fig. S1b, SI 1 presents Raman spectroscopy of the BDD film. The Raman spectrum reveals the presence of a sharp peak at 1333 cm^{-1}, which corresponds to the diamond zone centre optical phonon peak associated with sp^3 carbon [1,2]. The diamond peak is overlapping with a signal originating from a disordered *D*-band peak of non-diamond carbon occurring at *ca.* 1355 cm^{-1}. The presence of a third peak at 1509 cm^{-1} corresponding to the *G*-band of graphite also indicates sp^2–hybridised carbon [3]. In crystalline graphite the *G*-band peak is observed at 1575 cm^{-1} [1], however, the presence of amorphous carbon could shift the signal toward smaller values [3,4].

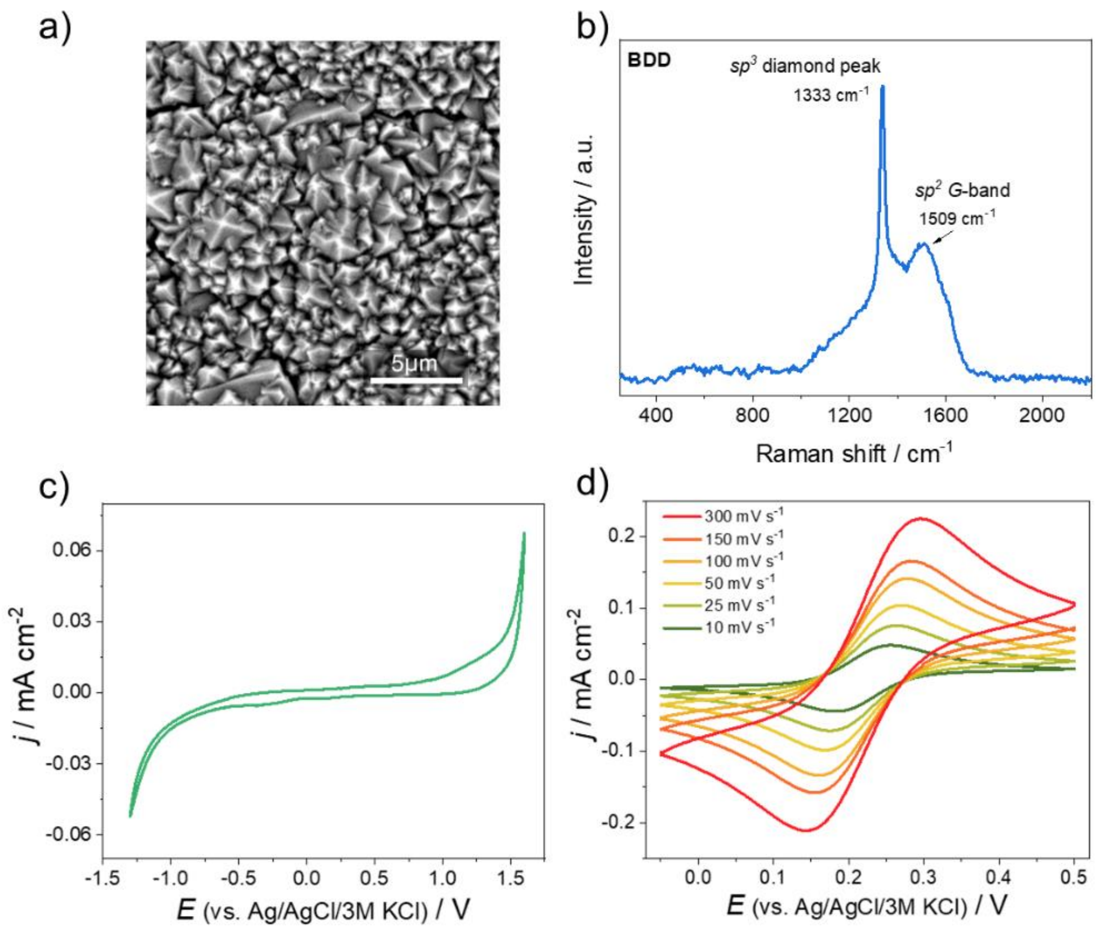

Fig. S1. Properites of BDD electrode: a) scanning electron micropscopy image with magnitude of × 10 000, b) Raman spectroscopy,) solvent window recorded in PBS, d) cyclic voltammetry curves recorded in 1 mM $[Fe(CN)_6]^{3-/4-}$ in 1 M KCl as a function of the scan rate.

The electrochemical behaviour of BDD electrode was evaluated using cyclic voltammetry (Fig. S1c–d). The solvent window (SW) defined by a geometric current density of ± 0.025 mV cm^{-2} in PB solution was featureless and wide with SW equal to 2.67 V (ν = 100 mV s^{-1}). Electrochemical kinetic behaviour was characterized using $[Fe(CN)_6]^{3-/4-}$ redox system. Figure S1d presents a cyclic voltammograms for BDD immersed in 1 mM $[Fe(CN)_6]^{3-/4-}$ in 1 M KCl at the different scan rates (from 10 to 300 mVs^{-1}) in the potential range of –0.05 to +0.50 V *vs.* Ag/AgCl/3M KCl. As can be seen, well-defined oxidation and reduction peaks can be observed. The separation between the oxidation and reduction peak potentials (ΔE_p) value obtained for 50 mVs^{-1} was equal 95.6 ± 4.4 mV, the oxidation density current j_{ox} was equal 102.9 ± 5.6 μA cm^{-2}, and anodic to cathodic density current ratio $j_{red} : j_{ox}$ is close to 1 (detailed values are gathered in Table S1). Moreover, the BDD electrode shows a linear dependence of the current *vs.* the square root of scan sweep described by Randles-Sevcik Equation (Figure S2a, SI 1), which suggests that the oxidation/reduction reactions of the redox mediator are

diffusion-controlled. [5]. The anodic and cathodic regression lines are nearly symmetrical [6]. Furthermore, the logarithm of the anodic peak current densities *vs*. the logarithm of the scan rate is also linear (Fig. S2b, SI 1), with a slope of 0.435. For the pure diffusional-controlled process the slope should be 0.5 [7–9]. Thus, the acquired result show that electrochemical reaction for $[Fe(CN)_6]^{3-/4-}$ redox system are governed by the diffusion-control mechanism.

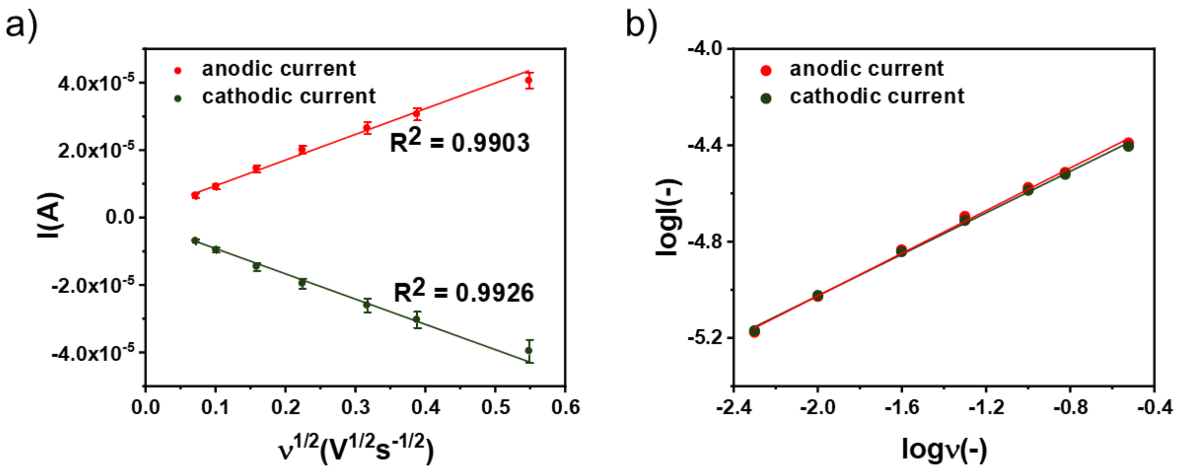

Fig. S2. a) Dependence of the peak current on the square root of a scan rate, b) the relation of the logarithm of peak current versus the logarithm of the scan rate recorded on BDD electrodes immersed in 1 mM $K_3[Fe(CN)_6]/K_4[Fe(CN)_6]$ in 1 M KCl.

Table S1. The estimated oxidation (j_{ox}) and reduction (j_{red}) density currents, and peak-to-peak separation values (ΔE_p) for various scan rates recorded on BDD electrodes immersed in 1 mM $K_3[Fe(CN)_6]/K_4[Fe(CN)_6]$ in 1 M KCl.

v / mV s^{-1}	ΔE_p / mV	j_{ox} / µA cm^{-2}	j_{red} / µA cm^{-2}	$j_{red}:j_{ox}$
5	65.2 ± 1.7	33.9 ± 3.2	34.5 ± 2.0	1.02
10	71.3 ± 2.2	47.8 ± 3.8	48.3 ± 3.6	1.01
25	82.4 ± 2.8	74.8 ± 5.3	73.4 ± 6.1	0.98
50	95.6 ± 4.4	102.9 ± 5.6	98.9 ± 7.9	0.96
100	109.8 ± 4.2	135.8 ± 9.1	131.9 ± 9.8	0.97
150	117.5 ± 5.3	156.8 ± 9.1	153.6 ± 12.4	0.98
300	139.5 ± 5.3	207.7 ± 12.0	200.9 ± 17.5	0.97

SI 2: Chemicals and solutions

Characterisation of the TNT

Table S2. Selected properties of the TNT.

Parameter	TNT
**Aspect**	light yellow crystals
**Density [g cm^{-3}]**[1]	1.63 ± 0.01
**Melting point [°C]**[2]	80.4 ± 0.2
**Decomposition point [°C]**[2]	324.2 ± 0.8
**Purity [%]**[3]	98.6 ± 0.3
**Friction sensitivity [N]**[4]	>353
**Impact sensitivity [J]**[5]	15

[1] determined by gas pycnometer (Ultrapyc 1200e); [2] determined by DSC method with hermetic aluminium pans, the temperature of peak maximum (DSC Q250 TA Instruments); [3] determined by the HPLC method (LC-2030C 3D Shimadzu); [4] determined by Julius Peters apparatus; [5] determined by Kast apparatus.

Environmental matrices characterisation: A sample of treated wastewater (TWW) was collected from the municipal wastewater treatment plant (WWTP; 470,000 people equivalent) located in the northern Poland Pomeranian region. This is a conventional WWTP which includes a biological step (the Bardenpho process with simultaneous denitrification in the Carussel system). Marine water (MW) was taken from the Baltic Sea (Puck Bay). The basic characteristics of the non-spiked samples are presented in Table S3. Before the chemical analysis, the environmental samples of TWW and MW taken during the AO process were subjected to degassing by mixing on a multipoint stirrer (Variomag, POLY 15 KOMED, Thermofisher Scientific, USA) at 50 rpm for 10 min. Determination of chemical oxygen demand (COD), inorganic N compounds (N-NH_4^+, N-NO_3^-, N-NO_2^-), chloride (Cl^-), and other ions (SO_4^{2-}, S^{2-}, P-PO_4^{3-}) for the TWW and MW characteristic was performed using a DR 3900 spectrophotometer (Dr. Lange, GmbH, Germany). Twenty-day biochemical oxygen demand (BOD20) was obtained using the manometric respirometric BOD OxiTop® method. Total suspended solids (TSS), volatile suspended solids (VSS) and mineral suspended solids (MSS) were obtained using the gravimetric method accordingly to the American Public Health Association (APHA) methods [10]. Conductivity, pH, Oxidation Reduction Potential (ORP) and temperature were measured by a portable multi-parameter meter HL-HQ40d (multi, HACH, Germany).

Table S3. Treated wastewater and marine water characteristics [mean ± SD].

	Parameter	TWW	MW
**Organic matter characteristics**	CHZT / mg L^{-1}	312 ± 25	54.4 ± 9.3
	BZT_5 / mg L^{-1}	78.9 ± 7.2	nd
	TSS / mg L^{-1}	44 ± 4.1	nd
	MSS / mg L^{-1}	5 ± 0.3	nd
	VSS / mg L^{-1}	39 ± 5.8	nd
**Nitrogen forms**	$N\text{-}NH_4^+$ / mg L^{-1}	74.1 ± 0.9	0.21 ± 0.7
	$N\text{-}NO_3^-$ / mg L^{-1}	0.869 ± 0.02	0.304 ± 0.005
	$N\text{-}NO_2^-$ / mg L^{-1}	0.087 ± 0.004	1.33 ± 0.03
	TN / mg L^{-1}	75.2 ± 1.4	2.51 ± 0.07
	$N\text{-}NH_4^+$ / TN	0.99 ± 0.01	0.084 ± 0.001
**Phosphate forms**	$P\text{-}PO_4^{3-}$ / mg L^{-1}	7.67 ± 0.89	0.48 ± 0.02
	TP / mg L^{-1}	8.22 ± 0.7	0.53 ± 0.01
	$P\text{-}PO_4^{3-}$ / TP	0.93 ± 0.11	0.906 ± 0.07
**Other ions**	Cl^- / mg L^{-1}	86.6 ± 1.2	3880 ± 39
	SO_4^{2-} / mg L^{-1}	39.6 ± 2.2	554 ± 42
	S^{2-} / mg L^{-1}	0.094 ± 0.009	0.011 ± 0.001
**Basic physicochemical parameters**	pH	7.76 ± 0.1	6.21 ± 0.10
	ORP / mV	44 ± 1.8	206.2 ± 2.0
	Conductivity / mS cm^{-1}	1.63 ± 0.3	11.9 ± 1.1
**Selected micropollutants**	TNT / mg L^{-1}	<LOD	<LOD

Abbreviations: TSS –total suspended solids, MSS –mineral suspended solids, VSS – volatile suspended solids, TN – total nitrogen, TP – total phosphorus, nd – not detected; LOD – limit of detection.

Other chemicals characterisation: A 0.1 M phosphate buffer solution (PBS) was used (8.733 g of K_2HPO_4 (purity 99%, Sigma Aldrich) and 125 µL of H_3PO_4 (85 wt. % in H_2O, 99.99%, Sigma Aldrich) were dissolved in a 500-mL of purified water (ultrapure water, resistivity 18.2 MΩ cm at 25°C obtained via Direct-Q® Water Purification System) with the final pH of 7.4.

Chloride standard solution (from NaCl) 1000 mg Cl^- L^{-1} (Certipur®, Supelco) was used to prepare spiked PBS.

Chromatography standards of TNT was supplied from Accu Standard, InC. (USA). Standards of 4-NT, 1,3-DNB, 2,6-DNT, 2,4-DNT, and TNB were synthesised in the Department of Explosive Materials of the Military University of Technology. The purity of the compounds was determined by GC-MS and was above 98% for all compounds. Standards of 4-A-2,6-DNT and 2-A-4,6-DNT were supplied from Supelco (Bellefonte, PA, USA). The purity of these standards exceeded 97% (validated with gas chromatography-mass spectrometry). Acetonitrile, chloroform, methanol, sodium chloride, magnesium sulphate, formic acid, and acetic acid were purchased from Sigma Aldrich (Milwaukee, USA) all reagent grades.

SI 3: Anodic oxidation process control

Chemical analysis results

Table S4. Process control parameters during electrooxidation of TNT removal from the environmental samples using BDD electrodes under the current density = 50 mA cm^{-2}.

Time / h	pH / -	Cond. / mS cm^{-1}	ORP / mV	i / A	U / V	Q / Ah dm^{-3}	ECon / kWh m^{-3}
**0.1M_PBS**							
0	7.77	15.14	334.2	0.5	9.2	-	-
1	7.79	-	-397.8	0.5	8.9	1	8.9
2	7.71	-	-365.6	0.5	8.9	2	17.8
3	7.80	-	-352.0	0.5	8.9	3	26.7
4	7.53	-	-303.8	0.5	8.9	4	35.6
5	7.50	-	-291.5	0.5	8.9	5	44.5
6	7.20	-	-309.4	0.5	8.9	6	53.4
7	7.21	-	-344.8	0.5	8.9	7	62.3
8	7.06	15.12	-344.5	0.5	8.9	8	71.2
**0.1M_PBS_100mgCl**							
0	7.01	15.16	275.9	0.5	9.4	-	-
1	8.04	-	-305.8	0.5	9	1	9
2	8.05	-	-315.0	0.5	9	2	18
3	7.76	-	-294.3	0.5	9.2	3	27.6
4	7.64	-	-299.8	0.5	9.1	4	36.4
5	7.41	-	-306.2	0.5	9.1	5	45.5
6	7.52	-	-320.6	0.5	8.9	6	53.4
7	7.33	-	-269.3	0.5	8.9	7	62.3
8	7.34	15.46	-301.5	0.5	9	8	72
**0.1M_PBS_200mgCl**							
0	6.85	15.9	87.0	0.5	8.9	-	-
1	7.04	-	-241.3	0.5	7.7	1	7.7
2	7.41	-	-227.2	0.51	8	2.04	16.32
3	7.24	-	-203.9	0.51	8.2	3.06	25.09
4	7.27	-	-200.4	0.51	8.6	4.08	35.09
5	7.18	-	-194.4	0.51	8.7	5.1	44.37
6	7.18	-	-211.1	0.5	8.6	6	51.6
7	7.21	-	-235.3	0.5	8.7	7	60.9
8	7.38	15.78	-230.9	0.5	8.6	8	68.8
**TWW**							
0	7.40	1.476	27.9	0.5	45.3	-	-
1	7.08	-	-291.3	0.5	35.4	1	35.4
2	6.98	-	-313.0	0.5	35.2	2	70.4
3	6.78	-	-303.7	0.5	36.3	3	108.9
4	6.84	-	-287.1	0.5	38	4	152
5	7.00	-	-292.9	0.5	36.9	5	184.5
6	7.02	-	-296.3	0.5	36.8	6	220.8
7	7.18	-	-299.5	0.5	36.3	7	254.1
8	7.16	1.829	-301.4	0.5	35.2	8	281.6

Time / h	pH / -	Cond. / mS cm^{-1}	ORP / mV	*i* / A	*U* / V	Q / Ah dm^{-3}	ECon / kWh m^{-3}
			**MW**				
0	5.81	11.3	545.5	0.5	11.5	-	-
1	2.88	-	1068	0.49	10.2	0.98	10.0
2	3.15	-	1109.2	0.5	10	2	20.0
3	3.18	-	1056.1	0.5	9.9	3	29.7
4	3.40	-	928.0	0.5	10.1	4	40.4
5	5.25	-	851.6	0.5	10.2	5	51.0
6	6.76	-	743.4	0.5	10.5	6	63.0
7	7.01	-	777.6	0.49	10.5	6.86	72.0
8	6.72	10.2	782.1	0.5	10.6	8	84.8

Abbreviations: *i* – current; *U* – voltage; *Q* – specific electrical charge; *ECon* – Energy consumption.

HPLC-PDA and GC-MS/MS parameters and results

High-performance liquid chromatography with a photodiode array detector, HPLC-PDA, (Prominence-i LC-2030 3D plus, Shimadzu, Japan) was used to analyse the effectiveness of TNT removal during the AO process. Data acquisitions were performed using Lab Solution software (Shimadzu, Japan). A Kinetex LC 18 column, 250 mm x 4.6 mm, 5 μm fill diameter (Phenomenex, DE) with a flow rate of 1.0 mL min^{-1} was used to separate analytes peaks. The samples were analysed in a gradient system using the mobile phase with a flow rate 1.0 mL min^{-1} (phase A: acetonitryle trifluoroacetic acid) (1 / 0.1) (v / v); phase B: water: trifluoroacetic acid (1 / 0.1) (v / v). The injection volume of injected samples was 10 μL.

A gas chromatograph (Thermo Trace 1310) equipped with tandem mass spectrometer TSQ 8000 Evo (Thermo Fisher Scientific, Bremen, Germany) was used for gas chromatography-tandem mass spectrometry (GC-MS/MS) investigation. Mass Hunter B.07.00 (Agilent Technologies) software was used for data acquisition. The measurements were conducted using a TraceGOLD TG-5SilMS 30 m × 0.25 mm × 0.25 μm capillary column (Thermo Fisher Scientific). The analysis was performed using the following temperature program: the column was heated from 100 to 250°C at a rate of 10°C min^{-1}, whereas the maximum temperature was kept for 12 min. The injector and transfer line temperatures were 270°C, while the ion source temperature was 250°C. The He carrier gas flow rate was 1 mL min^{-1}. During the analysis, the injector was in splitless mode, and its assigned time of operation was 2 min. The mass spectrometer was operated in electron impact ionisation mode at 70 eV. The detector operated at full scan mode with mass range from m/z 40 to 500 and scan time 180 ms, and for quantitative analysis selected ion monitoring (SRM) was used (SRM transitions in gathered in Table S5). Before the analysis the samples were first pre-treated in order to determine TNT

and its degradation products. For this purpose, 2 mL of the sample was transferred to a 10-mL glass vial with 2 mL of chloroform and 0.1 g of NaCl. Next, the solution was shaken for 5 min and centrifuged at 2500 rpm for 10 min. The separated organic phase was transferred to vial containing 0.5 g of $MgSO_4$, and was shaken for 60 min at the rate of 1500 rpm then decanted from the magnesium sulphate sediment to the GC 2 mL vial and analysed by GC-MS/MS in full scan mode and SRM mode.

Table S5. SRM transitions used during the analysis of TNT and its degradation products by GC-MS/MS.

Compound	Parent ion / m/z	Transition precursor ion / product ion	Collision cel energy / eV	Type of transition
4-nitrotoluene	137	137/137	0	Q
		137/107	3	q
		137/91	13	q
1,3-dinitrobenzene	168	168/168	0	Q
		168/122	8	q
		168/75	22	q
2,6-dinitrotoluene	165	165/165	0	Q
		165/148	9	q
		165/90	16	q
2,4-dinitrotoluene	165	165/165	0	Q
		165/119	6	q
		165/118	10	q
1,3,5-trinitrobenzene	213	213/213	0	Q
		213/167	8	q
		213/120	21	q
2,4,6-trinitrotoluene	210	210/210	0	Q
		210/193	10	q
		210/164	6	q
2,6-dinitro-4-aminotoluene	197	197/197	0	Q
		197/180	5	q
		197/78	25	q
	180	180/180	0	Q
		180/163	9	q
		180/105	11	q
2,4-dinitroaniline	183	183/183	0	Q
		183/153	6	q
		183/107	15	q
4,6-dinitro-2-aminotoluene	197	197/197	0	Q
		197/180	3	q
		197/133	14	q
	180	180/180	0	Q
		180/133	8	q
		180/105	19	q

Table S6. Limit of detection and quantification for GC-MS/MS in selected reaction monitoring mode used for the analysis of TNT and its degradation products.

No.	Analyte	LOD / ng mL^{-1}	LOQ / ng mL^{-1}	CoV / %	Accuracy / %
1.	2,4,6-trinitrotoluene (TNT)	560	1698	2.8	100.5
2.	4-nitrotoluene (4-DNT)	0.6	1.9	5.5	98.7
3.	1,3-dinitrobenzene (1,3-DNB)	3.6	11.0	3.5	102.6
4.	2,6-dinitrotoluene (2,6-DNT)	2.0	6.1	1.5	100.7
5.	2,4-dinitrotoluene (2,4-DNT)	2.7	8.1	5.1	101.2
6.	1,3,5-trinitrobenzene (TNB)	34.9	106	5.0	101.1
7.	4-amino-2,6-dinitrotoluene (4-A-2,6-DNT)	4.7	14.2	7.1	103.1
8.	2-amino-4,6-dinitrotoluene (2-A-4,6-DNT)	13.1	39.7	7.3	102.5

Abbreviations: CoV – coefficient of variation; LOD – limit of detection; LOQ – limit of quantification.

SI 4: Fabrication and characterization of BDGNW electrodes

Fabrication of BDGNW electrodes: The electrodes were deposited on silicon wafers using a microwave plasma-enhanced chemical vapour technique (SEKI Technotron AX5400S, Japan). Carbon electrode was doped by using diborane (B_2H_6) as boron precursor. The boron to carbon ratios in the plasma were adjusted to 2,000 ppm. To maintain a temperature of approximately 700°C, an external heater was employed to heat the substrate, with the substrate temperature monitored using a thermocouple embedded in the substrate holder. BDGNW samples were produced with a growth time of 6 hours. Before the chemical vapor deposition growth, the silicon (100)-oriented substrates were seeded through spin-coating in a diamond slurry.

Characterisation of BDGNW electrodes: The physicochemical and electrochemical properties of the fabricated electrodes were shown in our previous work [11].

References:

1. Macpherson, J. V. A practical guide to using boron doped diamond in electrochemical research. *Phys. Chem. Chem. Phys.* **17**, 2935–2949 (2015).
2. Hutton, L. A. *et al.* Examination of the factors affecting the electrochemical performance of oxygen-terminated polycrystalline boron-doped diamond electrodes. *Anal. Chem.* **85**, 7230–7240 (2013).
3. Ferrari, A. & Robertson, J. Interpretation of Raman spectra of disordered and amorphous carbon. *Phys. Rev. B* **61**, 14095–14107 (2000).
4. Peña-Álvareza, M., del Corro, E., Langa, F., Baonza, V. G. & Taravillo, M. Morphological changes in carbon nanohorns under stress: a combined Raman spectroscopy and TEM study. *Rsc Adv.* **6**, 49543–49550 (2016).
5. Elgrishi, N. *et al.* A Practical Beginner's Guide to Cyclic Voltammetry. *J. Chem. Educ.* **95**, 197–206 (2018).
6. Aristov, N. & Habekost, A. Cyclic Voltammetry - A Versatile Electrochemical Method Investigating Electron Transfer Processes. *World J. Chem. Educ. Vol. 3, 2015, Pages 115-119* **3**, 115–119 (2015).
7. David K. Gosser Jr. *Cyclic Voltammetry Simulation and Analysis of Reaction Mechanisms*. (VCH, 1993).
8. Pierpaoli, M. *et al.* Simultaneous opto-electrochemical monitoring of carbamazepine and its electro-oxidation by-products in wastewater. *J. Hazard. Mater.* **419**, 126509 (2021).
9. Siuzdak, K. *et al.* Boron-Enhanced Growth of Micron-Scale Carbon-Based Nanowalls: A Route toward High Rates of Electrochemical Biosensing. *ACS Appl. Mater. Interfaces* **9**, 12982–12992 (2017).
10. APHA-AWWA-WEF. *Standard methods for the examination of water and wastewater*. (2005).
11. Dettlaff, A. *et al.* Electrochemical determination of nitroaromatic explosives at boron-doped diamond/graphene nanowall electrodes: 2,4,6-trinitrotoluene and 2,4,6-trinitroanisole in liquid effluents. *J. Hazard. Mater.* **387**, 121672 (2020).
